# Association Between Antibiotic Treatment of Leptospirosis Infections and Reduced Risk of Dementia: A Nationwide, Cohort Study in Taiwan

**DOI:** 10.3389/fnagi.2022.771486

**Published:** 2022-03-23

**Authors:** Pei-Chun Chao, Wu-Chien Chien, Chi-Hsiang Chung, Chih-Kang Huang, Hao-Ming Li, Nian-Sheng Tzeng

**Affiliations:** ^1^Department of Psychiatry, Tri-Service General Hospital, School of Medicine, National Defense Medical Center, Taipei, Taiwan; ^2^Department of Psychiatry, Tri-Service General Hospital, National Defense Medical Center, Taipei, Taiwan; ^3^Department of Medical Research, Tri-Service General Hospital, National Defense Medical Center, Taipei, Taiwan; ^4^School of Public Health, National Defense Medical Center, Taipei, Taiwan; ^5^Graduate Institute of Life Sciences, National Defense Medical Center, Taipei, Taiwan; ^6^Taiwanese Injury Prevention and Safety Promotion Association, Taipei, Taiwan; ^7^Department of Emergency Medicine, Tri-Service General Hospital, School of Medicine, National Defense Medical Center, Taipei, Taiwan; ^8^Department of General Surgery, Tri-Service General Hospital, School of Medicine, National Defense Medical Center, Taipei, Taiwan; ^9^Student Counseling Center, National Defense Medical Center, Taipei, Taiwan

**Keywords:** leptospirosis, antibiotic, dementia, time-dependent, risk

## Abstract

**Background:**

To explore the association between leptospirosis, the risk of dementia, and the potential protective role of antibiotic treatment.

**Methods:**

We conducted a retrospective cohort nationwide, population-based study, from Taiwan’s National Health Insurance Research Database (NHIRD). We enrolled 1,428 subjects aged 50 years or above, in the index year of 2000, which included those retrieved from the NHIRD record. Dementia diagnosis and incidence over 16 years follow-up was retrieved from the NHIRD records. The Fine and Gray survival analysis was used to determine the risk of dementia, and the results were presented as a sub-distribution hazard ratio (SHR) with a 95% confidence interval.

**Results:**

In the study period, 43 of the 357 leptospirosis patients developed dementia, as compared to 103 of the control group (930.90 vs. 732.49 per 10^5^ person-years). By the Fine and Gray survival analysis, the leptospirosis was associated with the risk of dementia, and the adjusted SHR was 1.357 (95% confidence interval [CI]: 1.213–1.519, *P* < 0.001), across 16-year of the follow-up period. To exclude the protopathic bias, the sensitivity analysis was conducted. This analysis revealed that the leptospirosis was associated with the increased risk of dementia, even after excluding the dementia diagnosis within the first year (adjusted SHR = 1.246, 95%CI: 1.114–1.395, *P* < 0.001) or within the first 5 years (adjusted SHR = 1.079, 95%CI: 1.023–1.152, *P* = 0.028), antibiotic treatment for leptospirosis was associated with the reduced risk of dementia (*P* = 0.001).

**Conclusion:**

Leptospirosis was associated with an increased risk for dementia, and antibiotic treatment was associated with a reduced risk. Further research will be necessary to explore the underlying mechanisms of this association.

## Introduction

In Taiwan, 4–8% of those aged 65 years or over have dementia (Sun et al., 2014), which is a heavy burden for the patients, their caregivers, and the community. Alzheimer’s dementia (AD) is the most common type of dementia, which is a progressive condition that principally affects the elderly. Even though the underlying etiology of AD is not known, there are extensive beta-amyloid (Aβ) deposits in the brain of individuals with no signs of cognitive impairment (Rodrigue et al., 2009). In addition, Aβ may play a role in response to several types of infection (Soscia et al., 2010; Moir et al., 2018). Several recent studies have focused on the possibility that infectious agents might be predisposed to the AD development (Itzhaki et al., 2016). Research has focused on pathogens, such as herpes viruses, yeasts, or bacteria, notably including spirochetes (Miklossy, 2011).

The spirochetes pathogens are generally acquired by the exposure to wild animal secretions or arthropod bites, which are the most prevalent spirochetosis worldwide, particularly in wet tropical and subtropical regions (Levett, 2001). A broad spectrum of clinical manifestations may occur in humans, ranging from subclinical infections and self-limited anicteric febrile illnesses to the severe and potentially fatal icteric disease including Weil’s disease. Antibiotics, especially penicillin, are the mainstay of treatment for suspected or confirmed cases of leptospirosis, and treatment with the appropriate antibiotics should be initiated immediately (Center for Disease Control and Prevention [CDCP], 2018). Although some infected individuals display fulminant disease, it is suspected that the chronic carriage in others can remain subclinical. The treatment of leptospirosis differs depending on the severity and duration of the symptoms at the time of presentation, and patients with mild, flu-like symptoms may require only symptomatic treatment.

A possible relationship between *Leptospira* infection and neuropsychiatric disorders has been previously discussed, including dementia, depression, mania, and psychosis (Marshall and Scrimgeour, 1978; Semiz et al., 2005; Chiu et al., 2019). Importantly, it was recently reported that patients with a history of leptospirosis were at a moderately increased risk of developing dementia (Chiu et al., 2019). In addition, since the allelic variants of Apolipoprotein E4 (*APOE4*) allele are a risk factor not only for AD (Strittmatter et al., 1993) but also for a disease caused by the herpes simplex virus (HSV)-1 (Burgos et al., 2006), human immunodeficiency virus (HIV)-1 (Burt et al., 2008), and bacteria including *Chlamydia pneumoniae* (Gerard et al., 2005), we hypothesize that there might be a link between leptospirosis infections and dementia. For this reason, we have conducted a retrospective cohort study so as to investigate the association between leptospirosis and dementia, and the role of the antibiotic treatment in the risk of dementia in the leptospirosis group.

## Materials and Methods

### Data Sources

The National Health Insurance (NHI) Program was launched in Taiwan in 1995, and as of June 2009, includes contracts with 97% of medical providers, up to 23 million beneficiaries, and covers more than 99% of the entire population (Ho Chan, 2010). Several previous studies have documented the details of this program (Chen et al., 2020; Tzeng et al., 2020; Wan et al., 2020). We used the Taiwan NHI Research Database (NHIRD) to investigate the association between leptospirosis diagnosis and dementia over a 16-year period (2000–2015) for all outpatients and hospitalizations recorded in the NHIRD.

Diagnosis of leptospirosis was performed according to the International Classification of Diseases, 9th Revision, Clinical Modification (ICD-9-CM) (Chinese Hospital Association [CHA], 2000). Typical clinical findings indicative of leptospirosis were confirmed in all cases by a culture of *Leptospira* spp. and/or serology (Chiu et al., 2019). For the culture, blood and urine collected during the first 10 days of the disease were sent for microbiological analysis. For the serological diagnosis, paired acute and convalescent sera were analyzed by a microscopic agglutination test (MAT). The MAT titer is obtained by incubating serial dilutions of a patient’s serum with different *Leptospira* serovars; the serovar that reacts most strongly is suggested to be the infecting serovar. A positive laboratory diagnosis of leptospirosis required one of the following two criteria: (i) positive culture isolation, and/or (ii) a fourfold rise in MAT titer between the acute phase and the convalescent phase and a titer ≥1:400 in a single serum. Laboratory studies were performed at the Taiwan Centers for Disease Control [TCDC] (2017).

Dementia diagnosis was performed by board-certified neurologists or psychiatrists according to the Diagnostic and Statistical Manual of Mental Disorders, 4th Edition and its text-revised edition (American Psychiatric Association [APA], 1994, 2000). Licensed medical records technicians review and verify the diagnostic coding before claiming reimbursements (Chen et al., 1995). The NHI Administration randomly reviews the records of 1 in 100 ambulatory care visits and 1 in 20 in-patient claims to verify the accuracy of the diagnoses (Ministry of Justice, 2019). Several studies have demonstrated the accuracy and validity of the diagnoses in the NHIRD (Cheng et al., 2011; Liang et al., 2011; Chou et al., 2013).

### Study Design and Sampled Participants

**FIGURE 1:**
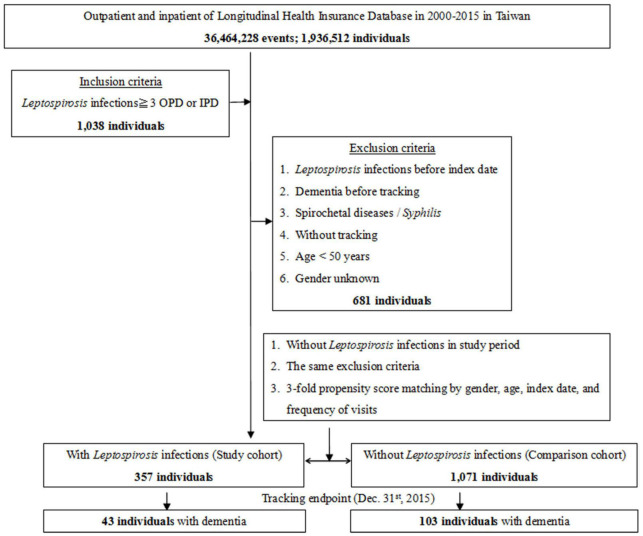
The flowchart of study sample selection.

This study was of a retrospective matched-cohort design. Patients with a history of leptospirosis, spirochetal disease, or syphilis, dementia diagnosis before the index date, who were aged <50 years, or where relevant information was missing were excluded from this study. Patients with leptospirosis were selected from January 1 to December 31, 2000, according to ICD-9-CM code 100 (100.0, 100.8, or 100.9). This included 357 patients first diagnosed with leptospirosis during the index year. A control group (*n* = 1038) matched for age and sex with no diagnosis of leptospirosis was also selected for study (Figure 1).

Covariates included gender, age group (50–64, ≥65 years), marital status, years of education, geographical area of residence (north, center, south, and east of Taiwan), urbanization level of residence area (levels 1 to 4), level of hospital as medical centers, regional and local hospitals, and insurance premiums (in New Taiwan Dollars [NT$]; <18 000, 18 000–34 999, ≥35 000). The urbanization level of residence was defined according to the total population and indicators of the level of development (Chiu et al., 2014). The comorbidities included diabetes mellitus, hypertension, hyperlipidemia, coronary artery disease, obesity, cancer, depressive disorder, bipolar disorder, anxiety disorder, alcohol usage disorder, substance usage disorder, sleep disorder, septicemia (ICD-9-CM codes listed in Supplementary Table 1). We also used the Charlson Comorbidity Index (CCI, scores of 0, 1, 2, 3, ≧4), which is the most widely used comorbidity index in the literature (Charlson et al., 1987; de Groot et al., 2003).

The antibiotics used in the treatment for the leptospirosis includeβ-lactams, cephalosporins, and doxycycline, and the data on the usage of these antibiotics were collected. The data of the defined daily dose (DDD) were obtained from the WHO Collaborating Centre for Drug Statistics Methodology^1^, and the duration of the usage of antibiotics was calculated by dividing the cumulative dosages by the DDD of the antibiotics. The duration of the antibiotic treatment is usually 7 days (Charan et al., 2013; Day, 2021), therefore, we divided the treatment durations as 7, 8–14, 15–21, 22–28, and >28 days, respectively.

### Outcome Measures

All the study participants were followed from the index date until the onset of dementia including Alzheimer’s dementia, vascular dementia, and other degenerative dementias, withdrawal from the NHI program, or the end of 2015. To ensure accuracy, each patient diagnosed with dementia was required to have made at least three outpatient visits during 1 year within the study period (Chiu et al., 2018).

### Statistical Analysis

All analyses were performed using the SPSS software version 22 (SPSS Inc., Chicago, IL, United States). χ^2^ and *t*-tests were used to evaluate the distributions of the categorical and continuous variables, respectively. The Fisher exact test for categorical variables was used to statistically examine the differences between the two cohorts. The Fine and Gray survival analysis was used to determine the risk of dementia, and the results were presented as a sub-distribution hazard ratio (SHR) with a 95% confidence interval (CI). The difference in the incidence of dementia between the study and control groups was estimated using the Kaplan–Meier method with log-rank test. A two-tailed *P*-value of <0.05 was considered so as to indicate the statistical significance.

## Results

### Sample Characteristics

The flowchart for enrollment of the leptospirosis patients and the controls is as presented in Figure 1. A total of 1428 patients were enrolled including 357 subjects with leptospirosis and 1071 controls without leptospirosis. The ratio of leptospirosis cohort and the control cohort was 1:3. These two cohorts were matched for age, sex, and index year (Figure 1 and Table 1). The prevalence of leptospirosis was 18.44 per 10^5^ (357 from an eligible population of 1 936 512) during the 16 years of follow-up; none of the control group received a diagnosis of leptospirosis during the study period. There were no differences between the groups in sex, age, marital status, or insurance premiums. In the leptospirosis cohort, there was a higher percentage of anxiety and septicemia and a lower percentage of coronary artery disease and cancer.

**TABLE 1:** Characteristics of study at the baseline.

*Leptospirosis* infections	With		Without		*P*
Variables	*n*	%	*n*	%	
Total	357	25.00	1,071	75.00	
Gender					0.999
Male	249	69.75	747	69.75	
Female	108	30.25	324	30.25	
Age (years)	63.35 ± 8.68		63.68 ± 8.67		0.532
Age groups (years)					0.999
50–64	220	61.62	660	61.62	
≧65	137	38.38	411	38.38	
Marital status					0.927
Without	159	44.54	480	44.82	
With	198	55.46	591	55.18	
Education (years)					0.903
<12	183	51.26	545	50.89	
≧12	174	48.74	526	49.11	
Insured premium (NT$)					0.005
<18,000	348	97.48	1,063	99.25	
18,000–34,999	9	2.52	6	0.56	
≧35,000	0	0.00	2	0.19	
Diabetes mellitus	54	15.13	149	13.91	0.600
Hypertension	57	15.97	216	20.17	0.087
Hyperlipidemia	8	2.24	38	3.55	0.298
Coronary artery disease	20	5.60	109	10.18	0.008
Obesity	2	0.56	0	0.00	0.062
Cancer	18	5.04	101	9.43	0.008
Depressive disorder	2	0.56	3	0.28	0.604
Bipolar disorder	0	0.00	2	0.19	0.414
Anxiety disorder	131	36.69	251	23.44	<0.001
Alcohol use disorder	0	0.00	6	0.56	0.346
Substance use disorder	0	0.00	2	0.19	0.414
Sleep disorder	4	1.12	4	0.37	0.113
Septicemia	51	14.29	28	2.61	<0.001
CCI_R					0.002
0	272	76.19	703	65.64	
1	67	18.77	258	24.09	
2	10	2.80	62	5.79	
3	6	1.68	27	2.52	
≧4	2	0.56	21	1.96	
Season					<0.001
Spring (Mar–May)	85	23.81	291	27.17	
Summer (Jun–Aug)	133	37.25	246	22.97	
Autumn (Sep–Nov)	78	21.85	220	20.54	
Winter (Dec–Feb)	61	17.09	314	29.32	
Location					<0.001
Northern Taiwan	89	24.93	439	40.99	
Middle Taiwan	59	16.53	280	26.14	
Southern Taiwan	43	12.04	284	26.52	
Eastern Taiwan	166	46.50	65	6.07	
Outlets islands	0	0.00	3	0.28	
Urbanization level					<0.001
1 (The highest)	81	22.69	404	37.72	
2	240	67.23	437	40.80	
3	13	3.64	68	6.35	
4 (The lowest)	23	6.44	162	15.13	
Level of care					<0.001
Medical center	257	71.99	396	36.97	
Regional hospital	96	26.89	380	35.48	
Local hospital	4	1.12	295	27.54	

*P: Chi-square/Fisher exact test on category variables and t-test on continue variables; NT$, New Taiwan Dollars; CCI_R, Charlson Comorbidity Index, dementia removed.*

### Kaplan–Meier Model for the Cumulative Incidence of Dementia

**FIGURE 2:**
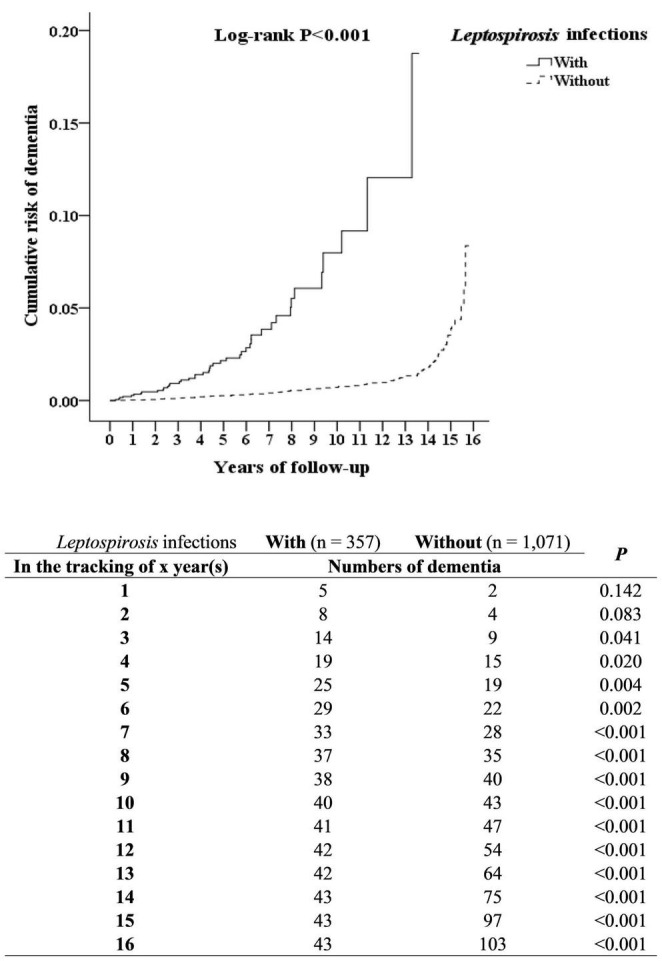
Kaplan–Meier for cumulative incidence of dementia aged 50 and over stratified by *Leptospirosis* infections with log-rank test.

In the overall study period, 43 of the 357 leptospirosis patients (930.90 per 10^5^ person-years) developed dementia as compared to 103 of the control group (*n* = 1071, 732.49 per 10^5^ person-years); the Kaplan–Meier analysis indicated that the difference was statistically significant (log-rank, *P* < 0.001, Figure 2).

### Hazard Ratio Analysis of Dementia in Patients With Leptospirosis

Fine and Gray’s competing risk model analysis revealed that the leptospirosis group was more likely to develop dementia (crude SHR = 1.350; 95% CI 1.211–1.496; *P* < 0.001). After adjustment for sex, age, monthly insurance premiums, urbanization level, geographic region, and comorbidities, the adjusted SHR over the 16-year period was 1.357 (95% CI 1.213–1.519; *P* < 0.001) (Table 2).

**TABLE 2:** Antibiotics usage and the risk of dementia by using Fine and Gray’s competing risk model.

	Competing risk in the model			
Variables	Adjusted SHR	95% CI	95% CI	*P*
*Leptospirosis* infections (reference: without)	1.357	1.213	1.519	<0.001
Severe infections	1.400	1.257	1.598	<0.001
Non-severe infections	1.326	1.202	1.483	<0.001
CCI_R = 1 (reference: CCI_R = 0)	3.098	1.124	8.012	0.003
CCI_R = 2 (reference: CCI_R = 0)	4.501	1.188	14.562	0.001
Winter (reference: spring)	6.532	1.397	33.012	0.001

*P: Chi-square/Fisher exact test on category variables and t-test on continue variables; SHR, sub-distribution hazard ratio; CI, confidence interval; Adjusted SHR, Adjusted variables listed in Table 1; CCI_R, Charlson Comorbidity Index, dementia removed.*

### The Risk and Sensitivity Analysis of Different Types of Dementia After Leptospirosis

As shown in Table 3, leptospirosis was associated with different types of dementia, including AD (adjusted SHR = 1.300 [95% CI: 1.162–1.455, *P* < 0.001]), VaD (adjusted SHR = 1.243 [95% CI: 1.111–1.391, *P* < 0.001]), or other degenerative dementia (adjusted SHR = 1.459 [95% CI: 1.305–1.634, *P* < 0.001]), respectively.

**TABLE 3:** Dementia types and sensitivity analysis using Fine and Gray’s competing risk model.

	*Leptospirosis* infections	Competing risk in the model			
Sensitivity test	Dementia subgroup	Adjusted SHR	95% CI	95% CI	*P*
Overall	Overall	1.357	1.213	1.519	<0.001
	AD	1.300	1.162	1.455	<0.001
	VaD	1.243	1.111	1.391	<0.001
	Other degenerative dementia	1.459	1.305	1.634	<0.001
In the first 1 year excluded	Overall	1.246	1.114	1.395	<0.001
	AD	1.220	1.091	1.366	0.001
	VaD	1.104	0.987	1.236	0.180
	Other degenerative dementia	1.352	1.208	1.513	<0.001
In the first 5 years excluded	Overall	1.079	1.023	1.152	0.028
	AD	1.117	1.058	1.195	0.009
	VaD	0.938	0.881	1.041	0.334
	Other degenerative dementia	1.142	1.076	1.222	0.002

*P: Chi-square/Fisher exact test on category variables and t-test on continue variables; PYs, Person-years; Adjusted SHR, Adjusted sub-distribution Hazard ratio: Adjusted for the variables listed in this table. CI, confidence interval; AD, Alzheimer’s dementia; VaD, vascular dementia.*

To exclude the protopathic bias, the sensitivity analysis was conducted. This analysis revealed that the leptospirosis was associated with the increased risk of dementia, even after excluding dementia diagnosis within the first year (adjusted SHR = 1.246, 95% CI: 1.114–1.395, *P* < 0.001) or within the first 5 years (adjusted SHR = 1.079, 95% CI: 1.023–1.152, *P* = 0.028). However, after excluding the diagnosis within the first and first 5 years, leptospirosis was associated with AD and other degenerative dementia, but not VaD.

### Subgroup Analysis for the Risk of Dementia After Leptospirosis

As shown in Table 4, the subgroup analysis revealed that the differential risk stratified by sex, age, marital status, educational level, monthly insurance premiums, comorbidities, urbanization and region of residence, CCI score, season, and level of medical care (possible exceptions include higher insured premiums). The subgroup analysis found that the leptospirosis patients with nearly all the covariates were associated with a higher risk of dementia, with the exceptions of insured premium of NT$18,000–34,999 and NT$ ≧35,000. In addition, the patients with or without most of these comorbidities were associated with a higher risk of dementia, with the exceptions of obesity, depression, bipolar disorder, alcohol usage disorder, other substance usage disorder, sleep disorder, and CCI as 3 and ≧4.

**TABLE 4:** Subgroup analysis stratified by variables listed in the table by using Fine and Gray’s competing risk model.

*Leptospirosis* infections	Competing risk in the model			
Stratified	Adjusted SHR	95% CI	95% CI	*P*
Total	1.357	1.213	1.519	<0.001
Male	1.429	1.278	1.600	<0.001
Female	1.219	1.089	1.364	<0.001
Age 50–64	1.266	1.131	1.417	<0.001
Age group≧65	1.464	1.308	1.638	<0.001
Married	1.332	1.190	1.491	<0.001
Not married	1.102	0.985	1.233	0.121
Education (years) < 12	1.328	1.187	1.487	<0.001
Education (years)≧12	1.507	1.347	1.687	<0.001
Insured premium (NT$) < 18,000	1.357	1.213	1.519	<0.001
Insured premium (NT$) 18,000–34,999	−	−	−	−
Insured premium (NT$) 35,000	−	−	−	−
Without DM	1.212	1.084	1.357	<0.001
With DM	1.664	1.487	1.862	<0.001
Without HTN	1.309	1.170	1.466	<0.001
With HTN	1.468	1.312	1.643	<0.001
Without Hyperlipidemia	1.357	1.213	1.519	<0.001
With Hyperlipidemia	−	−	−	−
Without CAD	1.285	1.148	1.438	<0.001
With CAD	1.883	1.683	2.107	<0.001
Without Obesity	1.357	1.213	1.519	<0.001
With Obesity	−	−	−	−
Without Cancer	1.246	1.114	1.394	<0.001
With Cancer	3.154	2.819	3.530	<0.001
Without Depression	1.357	1.213	1.519	<0.001
With Depression	−	−	−	−
Without Bipolar	1.357	1.213	1.519	<0.001
With Bipolar	−	−	−	−
Without Anxiety	1.246	1.114	1.395	<0.001
With Anxiety	1.459	1.305	1.634	<0.001
Without Alcohol use disorder	1.357	1.213	1.519	<0.001
With Alcohol use disorder	−	−	−	−
Without other substance use disorder	1.357	1.213	1.519	<0.001
With other substance use disorder	−	−	−	−
Without Sleep disorder	1.540	1.377	1.724	<0.001
With Sleep disorder	0.000	−	−	0.885
Without Septicemia	1.189	1.063	1.331	<0.001
With Septicemia	2.361	2.111	2.643	<0.001
CCI_R 0	1.183	1.057	1.324	<0.001
CCI_R 1	1.431	1.279	1.602	<0.001
CCI_R 2	3.438	3.073	3.848	<0.001
CCI_R 3	−	−	−	−
CCI_R≧4	−	−	−	−
Spring	1.141	1.020	1.277	0.030
Summer	1.226	1.096	1.372	<0.001
Autumn	1.490	1.332	1.668	<0.001
Winter	2.182	1.950	2.443	<0.001
**Urbanization level**				
Urbanization level 1 (the highest)	1.208	1.080	1.352	<0.001
Urbanization level 2	1.330	1.189	1.488	<0.001
Urbanization level 3	−	−	−	−
Urbanization level 4 (the lowest)	1.704	1.524	1.908	<0.001
Level of care Hospital center	1.454	1.299	1.627	<0.001
Level of care Regional hospital	1.274	1.139	1.426	<0.001
Level of care Local hospital	1.144	1.023	1.281	0.029

*P: Chi-square/Fisher exact test on category variables and t-test on continue variables; PYs, Person-years; Adjusted HR, Adjusted sub-distribution Hazard ratio: Adjusted for the variables listed in Table 1; CI, confidence interval; NT$, New Taiwan Dollars; CCI_R, Charlson Comorbidity Index, dementia removed.*

### Effect of Leptospirosis Antibiotic Treatment and Risk of Dementia

**FIGURE 3:**
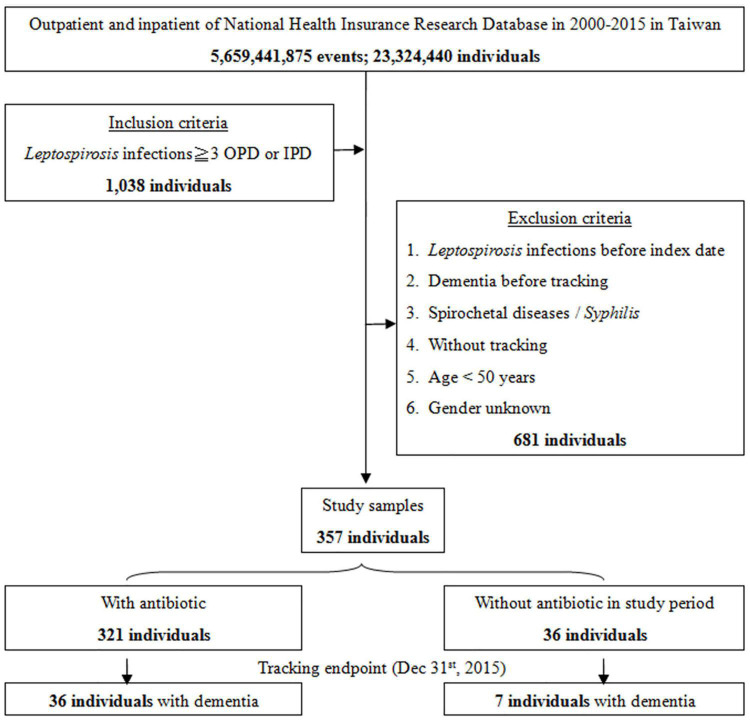
The flowchart of study sample selection with or without antibiotic treatment.

**FIGURE 4:**
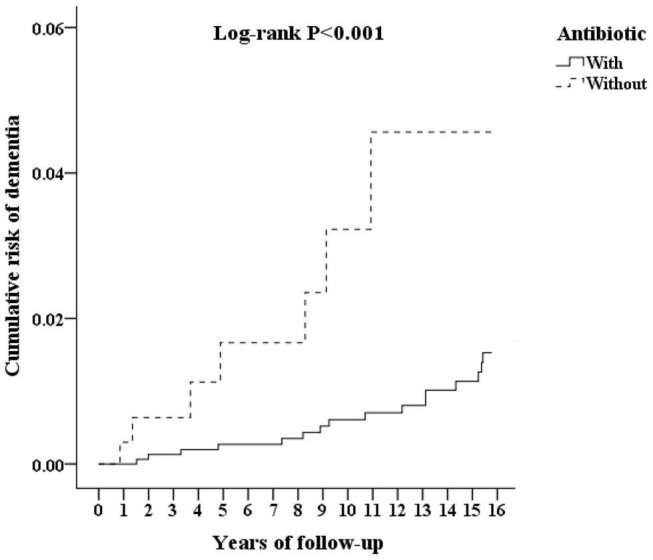
Kaplan–Meier for cumulative incidence of dementia aged 50 and over stratified by *Leptospirosis* infections with or without antibiotics, with log-rank test.

Although the majority of patients diagnosed with leptospirosis were recorded as having received antibiotic treatment (β-lactams, cephalosporins, and doxycycline), a small number were not treated. Figure 3 shows the flowchart of the study sample selection with or without antibiotic treatment. These subgroups differed in their risk of developing dementia. Of the leptospirosis patients with antibiotic treatment, 36 of 321 (916.52 per 10^5^ person-years) developed dementia compared to seven of 36 (938.04 per 10^5^ person-years) patients without antibiotic treatment; the difference was statistically significant in the Kaplan–Meier analysis (log-rank, *P* < 0.001; Figure 4). The risk of dementia development was lower in patients treated for a longer time or with higher antibiotic dosages (adjusted SHR = 0.685, 95%CI: 0.534–0.950, *P* = 0.001, Table 5).

**TABLE 5:** Percentage, duration, percentage of decrease of risk by days, and adjusted sub-distribution hazard ratios of antibiotic among *Leptospirosis* infections patients.

Antibiotic					With *vs.* without antibiotic (*Reference*)		
Type	*n*	%	Duration (days)		Competing risk in the model		
			Mean	SD	*n*% decrease of risk by day	Adjusted SHR (95% CI)	*P*
Overall	321		25.33	22.20	1.24	0.685 (0.534–0.950)	0.001
Beta-lactam	113	35.20	26.71	23.89	1.16	0.689 (0.542–0.962)	0.009
7 days	11	3.43	7.00	0.00	3.01	0.789 (0.604–1.102)	0.152
8–14 days	19	5.92	12.12	10.34	1.45	0.824 (0.608–1.189)	0.288
15–21 days	23	7.17	18.65	15.25	1.47	0.725 (0.592–0.978)	0.024
22–28 days	29	9.03	25.27	23.21	1.44	0.635 (0.489–0.929)	<0.001
>28 days	31	9.66	49.98	47.72	0.83	0.584 (0.415–0.827)	<0.001
Cephalosporin	130	40.50	24.73	21.17	1.33	0.672 (0.513–0.955)	0.001
7 days	21	6.54	7.00	0.00	3.54	0.752 (0.586–1.067)	0.134
8–14 days	18	5.61	12.35	10.25	1.34	0.835 (0.712–1.234)	0.294
15–21 days	30	9.35	18.51	15.11	1.63	0.698 (0.526–0.959)	0.007
22–28 days	25	7.79	25.98	23.72	1.38	0.641 (0.502–0.952)	0.001
>28 days	36	11.21	45.58	42.25	0.88	0.599 (0.442–0.863)	<0.001
Doxycycline	119	37.07	24.67	21.71	1.29	0.681 (0.494–0.938)	<0.001
7 days	20	6.23	7.00	0.00	3.26	0.772 (0.594–1.083)	0.148
8–14 days	18	5.61	12.13	10.98	1.31	0.841 (0.726–1.248)	0.304
15–21 days	25	7.79	18.72	15.34	1.51	0.718 (0.555–0.964)	0.012
22–28 days	25	7.79	25.19	23.08	1.47	0.629 (0.472–0.915)	<0.001
>28 days	31	9.66	47.72	45.97	0.86	0.588 (0.421–0.834)	<0.001

*P: Chi-square/Fisher exact test on category variables and t-test on continue variables; Adjusted SHR, Adjusted sub-distribution Hazard ratio: Adjusted for the variables listed in Table 1; CI, confidence interval; NT$, New Taiwan Dollars.*

### Time-Dependence of Risk of Dementia Development

Patients with a diagnosis of leptospirosis showed an overall increased risk of dementia development over the 16-year follow-up period (adjusted SHR = 1.357). However, the SHR evolved with time. The greatest differential was in the years immediately following the leptospirosis development, and the cumulative unnormalized relative incidences of dementia development were 3.96 at 6 years, 2.61 at 11 years, and 1.25 at 16 years. Cumulative SHR values adjusted for sex, age, monthly insurance premium, urbanization level, geographic region, and comorbidity were 1.296 at 6 years, to 1.331 at 11 years, to 1.357 at 16 years. Linear regression predicts that the SHR for dementia development between the two groups declines to 1.0 at 18–19 years following the leptospirosis development (Table 6).

**TABLE 6:** Factors of dementia by using Fine and Gray’s competing risk model.

	*Leptospirosis* infections (reference: without)							
Tracking period	Crude SHR	95% CI	95% CI	*P*	Adjusted SHR	95% CI	95% CI	*P*
Overall (In 16-year tracking)	1.647	1.315	2.103	<0.001	1.357	1.213	1.519	<0.001
In 1-year tracking	1.486	0.867	2.085	0.106	1.225	0.645	1.402	0.267
In 6-year tracking	1.569	1.047	2.117	0.002	1.296	1.030	1.426	0.009
In 11-year tracking	1.629	1.211	1.997	<0.001	1.331	1.176	1.798	<0.001

*P: Chi-square/Fisher exact test on category variables and t-test on continue variables; Adjusted HR, Adjusted sub-distribution Hazard ratio: Adjusted for the variables listed in Table 1; CI, confidence interval.*

### The Interaction Term Analysis Between Anxiety and Leptospirosis

Table 7 shows the subgroup analysis for the factors of dementia using the Fine and Gray’s competing risk model. The adjusted SHR of leptospirosis with anxiety was 2.806 (95% CI: 2.407–3.305, *P* < 0.001) and the adjusted SHR of leptospirosis without anxiety was 1.237 (95% CI: 1.095–1.386, *P* < 00.001) The *P*-values of the interaction term analysis of anxiety × leptospirosis was 0.001 in the non-competing risk model and <0.001 in the competing risk model.

**TABLE 7:** The interaction between *Leptospirosis* infections and anxiety for the risk of dementia.

*Leptospirosis* infections	Anxiety	Adjusted SHR	95% CI	95% CI	*P*
Without	Without	Reference			
Without	With	1.354	1.288	1.518	<0.001
With	Without	1.237	1.095	1.386	<0.001
With	With	2.806	2.407	3.305	<0.001

*Adjusted SHR, Adjusted sub-distribution hazard ratio: Adjusted for the variables listed in Table 1; CI, confidence interval. Interaction term (Joint Effect): Leptospirosis infections × Anxiety, P < 0.001.*

## Discussion

### Association Between Leptospirosis, Antibiotic Treatment, and the Risk of Dementia

We have reported that the individuals with a previous diagnosis of leptospirosis had an increased risk of developing any type of dementia, including AD and VaD (*P* < 0.001). When individuals with a diagnosis of dementia within the first year or the first 5 years were excluded, the leptospirosis patients were still associated with an increased risk of AD and related dementias. The results of our study (adjusted SHR = 1.357; 95% CI 1.213–1.519; *P* < 0.001; 16 years follow-up) are comparable to the conclusions of an earlier study on leptospirosis patients (adjusted SHR = 1.89; 95% CI 1.72–2.08; 10 years follow-up) (Chiu et al., 2019). In addition, leptospirosis was associated with AD, VaD, and other degenerative dementia. We also reported that treatment of the leptospirosis patients with antibiotic medications, such as β-lactams, cephalosporin, and doxycycline, for the first time, was associated with a reduced risk of dementia (adjusted SHR = 0.685) in a dose-dependent manner (*P* = 0.001).

The adjusted SHR increased from the sixth year and, as the Linear regression predicts, declined after the 16-years of follow-up (Table 6). This finding might hint that the risk of dementia would be higher between six and 16 years after the leptospirosis.

In the leptospirosis groups, there was a significant higher incidence of anxiety disorders (*N* = 131, 36.69%) as compared to the control group (*N* = 251, 23.44%). As previously reported, anxiety has been associated with all types of dementia (Santabarbara et al., 2020). Thus, we have conducted an interaction term analysis, which revealed an interaction between anxiety and leptospirosis in the contribution of the risk of dementia (Table 7).

### Possible Mechanisms of Association Between Leptospirosis and the Risk of Dementia

The underlying mechanisms of the association between leptospirosis infections and dementia remain unclear. Nonetheless, inflammatory changes in the brain have been reported in studies on the pathogenesis of dementia (Daulatzai, 2016; Stefaniak and O’Brien, 2016). Previous researchers have found that infectious diseases, such as hepatitis C, viral infection, Helicobacter pylori infection, cytomegalovirus infections, chronic osteomyelitis, or even sepsis, were associated with an increased risk of dementia (Barnes et al., 2015; Kao et al., 2015). Other antibiotic-susceptible bacterial species were widely present in both the control and AD brain (Branton et al., 2013; Emery et al., 2017). In 2019, Chiu first reported that patients with leptospirosis were associated with higher risks of dementia, pointing out the role of endothelial damage and vascular hypo-perfusion in subsequent chronic diseases such as dementia (Chiu et al., 2019).

*Leptospira* may cause cytokine storm, endothelial damage, vascular hypo-perfusion, and subsequent organ failure (Cagliero et al., 2018). AD is the most common cause of dementia. The major hypothesis on the etiology of AD is the amyloid cascade hypothesis (Barage and Sonawane, 2015). In addition, there is also growing evidence indicating that vascular dysfunction plays a pivotal role in the development of AD, and several vascular risk factors, such as hypertension, atherosclerosis, hyperlipidemia, and stroke, are associated with AD-type dementia (Launer et al., 2000; Fratiglioni et al., 2007). The significant association between vascular diseases and AD suggests that dementia associated with AD may be caused by vascular mechanisms.

However, the specific role of spirochetes is uncertain. For *Leptospira* spp., only 1.4% of United States army soldiers deployed in disease-endemic countries, and 2.5% of United States veterinarians, were reported to be seropositive (Lettieri et al., 2004; Whitney et al., 2009). In the United Kingdom, fewer than 100 cases of acute leptospirosis were recorded each year (Forbes et al., 2012). In addition, serological testing may not detect many individuals infected with *Borrelia* spp. (Lloyd and Hawkins, 2018). Several studies have found that, in the countries closer to the equator where *Leptospira* spp. seroprevalence rates closer to 40% (Gonwong et al., 2017; Garba et al., 2018; Sohail et al., 2018). Therefore, the role of the difference of seroprevalence of the *Leptospira* spp. in different countries might well need to be further studied.

### Possible Mechanisms of Association Between Antibiotics and the Lower Risk of Dementia in Patients With Leptospirosis

We also found that the antibiotic treatment was associated with a lower risk of dementia in patients with leptospirosis infections, and the adjusted SHR was 0.685. The leptospirosis-infected subjects treated with the antibiotics of beta-lactam, cephalosporin, and doxycycline showed a decreased risk of dementia, when compared to the group without antibiotic medications. Moreover, once the treatment duration was more than 14 days, the risk of dementia decreased subsequently with the treatment duration prolonged.

The role of the antibiotic treatment of leptospirosis infections for the prevention of dementia has not, as yet, been studied. The association between antibiotic usage and Alzheimer’s disease had only been studied in animal models before. Ceftriaxone may restore the glial glutamate transporter and further ameliorate tau pathology, and the cognitive decline in Alzheimer’s disease (Zumkehr et al., 2015). Neuroinflammation is a chronic event whose perpetuation leads to the continuous release of pro-inflammatory cytokines, promoting neuronal cell death and gross brain atrophy. Doxycycline emerged as a promising preventive strategy in prion diseases and gave compelling pre-clinical results in mouse models of AD against Aβ oligomers and neuroinflammation (Balducci and Forloni, 2019). Our present study might be the first to report on the role of antibiotic medication treatment in attenuating the risk of developing dementia for patients with leptospirosis infections in a nationwide, population-based study. However, in the leptospirosis group, only 10.1% (36 in 357) did not receive antibiotic treatment. This suggests that further study is needed so as to clarify whether antibiotic usage plays a role in reducing the risk of dementia for leptospirosis-infected patients.

### Strengths of This Study

The present study has several strengths: First, we used Taiwan’s NHIRD, which is a valuable resource to cover a nationwide population, to address this issue. Second, one previous study has demonstrated the accuracy and validity of several diagnoses of psychiatric disorders in the NHIRD, including major depressive disorder, schizophrenia, and dementia (Wu et al., 2020). Besides, the in-hospital licensed medical records technicians and NHI Administration would have verified the diagnoses in the claims dataset (Hsieh et al., 2019; Ministry of Justice, 2019), for the diagnosis. Third, previous studies have also demonstrated the concordance between Taiwan’s National Health Survey and the NHIRD on a variety of diagnoses (Wu et al., 2014), medication usage (Wu et al., 2014), and health system utilization (Yu et al., 2009; Wu et al., 2014).

### Limitations of This Study

This study has several limitations. First, regarding the antibiotic treatment, only 10.1% (36 of 357) of the leptospirosis group did not receive antibiotic treatment, and of these only seven went on to develop dementia. Although our results point to statistical significance, further investigations with a larger number of subjects will be essential to clarify whether the antibiotic treatment truly plays a role in reducing the risk of dementia for the leptospirosis-infected patients. Second, patients with dementia were identified using the insurance claims data, but data on severity or stage were not available, and we could only estimate the treatment durations of each antibiotic medication by dividing the cumulative doses of individual medications by the defined daily dose. Third, other confounding factors such as genetics and dietary factors are also not included in the NHIRD. Because no imaging findings or other laboratory data are included in the NHIRD, we relied on the professional diagnosis of dementia by board-certified psychiatrists or neurologists according to the ICD-9-CM codes from the NHIRD, and the leptospirosis cases that were presented as mild symptoms may not have been recorded and were thus not enrolled in this study. Nevertheless, because our analysis is based on a large cohort of patients (1428 subjects: 357 leptospirosis and 1071 controls) as well as internationally recognized diagnostic codes, and furthermore corroborates previous work on leptospirosis and AD risk (Chiu et al., 2019), our central results may be reliable – and raise three important issues concerning causation and the pathogen(s) that might be involved.

## Conclusion

Patients with leptospirosis infections have an increased risk of developing dementia, and the antibiotic treatment was associated with a diminished risk of dementia. These findings might well be considered as an indicator to clinicians caring for patients with leptospirosis infections. Further research will be necessary so as to explore the underlying mechanism(s) of this association.

## Data Availability Statement

The datasets on the study population that were obtained from the NHIRD (http://nhird.nhri.org.tw/en/index.html) are maintained in the NHIRD (http://nhird.nhri.org.tw/). The National Health Research Institutes (NHRI) is a nonprofit foundation established by the government. Only citizens of Taiwan who fulfill the requirements for conducting research projects are eligible to apply for the NHIRD. The use of the NHIRD is limited to research purposes only. Applicants must follow the Computer-Processed Personal Data Protection Act (https://dep.mohw.gov.tw/dos/lp-2506-113.html) and the related regulations of the National Health Insurance Administration and NHRI, and an agreement must be signed by the applicant and their supervisor upon application submission. All applications are reviewed for approval of data release.

## Ethics Statement

Since the identifiable database of individuals included in the NHIRD were all encrypted in order to protect individual privacy, the NHI Administration has given general approval for their data to be used in this research. Because the NHIRD has the advantage of providing a large-scale, longitudinal, reliable dataset, leading to extensive use for population-based researches in Taiwan, the Institutional Review Board of Tri-Service General Hospital was aware of this and approved the research to proceed, and also agreed that the benefit justified waiving the need for individual written informed consent in such a study (IRB No. 1-106-05-169).

## Author Contributions

P-CC and N-ST contributed to the study concept and design. W-CC, C-HC, and N-ST contributed to the acquisition of data. W-CC, C-HC, C-KH, H-ML, P-CC, and N-ST contributed to the analysis and interpretation of data. P-CC contributed to the drafting of the manuscript. N-ST contributed to the critical revision of the manuscript for important intellectual content. All authors contributed to the article and approved the submitted version.

## Conflict of Interest

The authors declare that the research was conducted in the absence of any commercial or financial relationships that could be construed as a potential conflict of interest.

## Publisher’s Note

All claims expressed in this article are solely those of the authors and do not necessarily represent those of their affiliated organizations, or those of the publisher, the editors and the reviewers. Any product that may be evaluated in this article, or claim that may be made by its manufacturer, is not guaranteed or endorsed by the publisher.

## Footnotes

1
https://www.whocc.no/
