# Supplementary material for: Association Between Antibiotic Treatment of Leptospirosis Infections and Reduced Risk of Dementia: A Nationwide, Cohort Study in Taiwan
Source: Front Aging Neurosci. 2022 Mar 23;14:771486. doi: 10.3389/fnagi.2022.771486 (PMC8985874; doi:10.3389/fnagi.2022.771486)
Supplement: Supplementary file 1 [file Table_1.DOCX]

| **Table S1. ICD-9-CM codes for study population, exclusions, outcomes, and comorbidities** | |
| --- | --- |
|  | **ICD-9-CM codes** |
| **Study population:** *Leptospirosis* infections | 100 |
| **Excluding** |  |
| *Syphilis* | 090-097 |
| Spirochetal diseases | 101-104 |
| **Outcomes:** Dementia | 290.0, 290.10-290.13, 290.20-290.21, 290.3. 290.40-290.43, 290.8-290.9, 331.0 |
| Alzheimer's disease | 331.0 |
| Vascular dementia | 290.4 |
| Other degenerative dementia | 290.x except 290.4 |
| **Comorbidities** |  |
| Diabetes mellitus | 250 |
| Hypertension | 401.1, 401.9, 402.10, 402.90, 404.10, 404.90, 405.1, 405.9 |
| Hyperlipidemia | 272 |
| Coronary artery disease | 410-414 |
| Obesity | 278 |
| Cancer | 140-208 |
| Depression | 296.2-296.3, 300.4, 311 |
| Bipolar disorders | 296.0-296.1, 296.4-296.9 |
| Anxiety disorders | 300, excluding 300.4 |
| Alcohol use disorders | 303, 305.00-305.03 |
| Substance use disorders | 304-305, excluding Alcohol use disorders |
| Sleep disorder | 307.4, 780.5 |
| Septicemia | 003.1, 036.2, 038, 790.7 |
| **ICD-9-CM = International Classification of Diseases,Ninth Revision, Clinical Modification** | |
